# Late-Onset Bowel Strangulation due to Reduction En Masse of Inguinal Hernia

**DOI:** 10.1155/2014/295686

**Published:** 2014-04-01

**Authors:** Ikuo Watanobe, Noritoshi Yoshida, Shin Watanabe, Toshirou Maruyama, Atsushi Ihara, Kuniaki Kojima

**Affiliations:** ^1^Department of Surgery, Asakusa Hospital, 1-10-12 Higashiasakusa, Taitou-ku, Tokyo 111-0025, Japan; ^2^Division of General Surgery, Juntendo University Nerima Hospital, 3-1-10 Takanodai, Nerima-Ku, Tokyo 177-8521, Japan

## Abstract

Incarcerated inguinal hernia is often encountered by surgeons in daily practice. Although rare, hernial reduction en masse is a potential complication of manual reduction of an incarcerated hernia. Manual reduction was performed in a case of Zollinger classification type VII (combined type) hernia in which the indirect hernia portion included an incarcerated small intestine. This procedure caused hernial reduction en masse, but this went unnoticed, and the remaining portion of the direct hernia in the inguinal region was treated surgically by the anterior approach. Because the incarcerated small bowel that had been reduced en masse was not completely obstructed, the patient's general condition was not greatly affected, and he was able to resume eating. Twenty days after surgery, he developed sudden abdominal pain as a result of gastrointestinal perforation. When performing manual reduction of an incarcerated hernia in cases after self-reduction over a long period, the clinician should always be aware of the possibility of reduction en masse.

## 1. Introduction

Hernial reduction en masse is a rare condition in which the hernial sac is returned to the properitoneal space along with incarcerated bowel during reduction. It can be defined as reduction of the hernia sac together with its intestinal contents so that the bowel still remains incarcerated. This condition requires surgical reduction and treatment to release strangulation. A case of small bowel perforation following reduction en masse that went unnoticed when a recurrent left inguinal hernia was manually reduced is reported.

## 2. Case Presentation

The patient was a 75-year-old man. He had undergone surgery for a left inguinal hernia 23 years earlier, but the details were unknown.

Four years earlier, the left inguinal hernia recurred and underwent repeated prolapse, spontaneous rectification, and self-reduction. Since last year, the hernia occasionally became incarcerated. On each occasion, the patient was treated by manual reduction as an outpatient, but he did not wish to undergo surgery, and the situation continued as it was. Over the previous month, the hernia frequently became incarcerated, and the patient visited our hospital as usual for manual reduction of the incarceration. This time the patient agreed to surgery and was admitted and treated surgically on the same day. Physical findings were as follows: height 170 cm, weight 70 kg, and temperature 36.6°C. The left lower abdomen was slightly tender, but there were no symptoms of peritoneal irritation such as rebound tenderness or guarding. After hernial reduction, neither inguinal region had any palpable swelling, and chest findings were normal.

Blood test findings were WBC 8600/*μ*L, Hb 10.4 g/dL, and CRP 0.19 mg/dL, indicating mild anemia and no inflammatory response. Abdominal X-ray on admission showed a dilated small bowel and niveau formation, indicating bowel obstruction. Pelvic CT revealed that the hernia had prolapsed into the left scrotum and the small bowel was dilated. There was no ascites.

**Figure 1:**
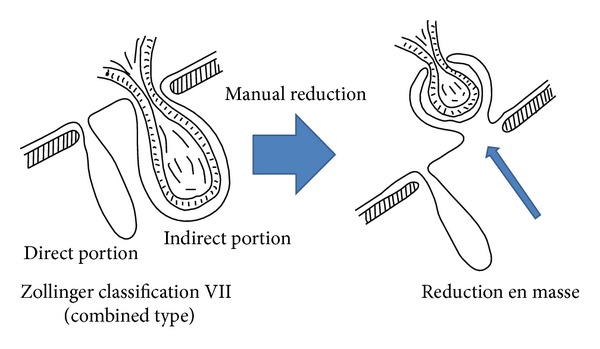
Our case illustration. It is a recurrent left inguinal hernia of Zollinger classification type VII (combined type). The indirect hernia portion includes an incarcerated small intestine. The indirect portion of reduction en masse occurred by manual reduction and the remaining portion of the direct hernia was treated surgically at first.

The first operation used the anterior approach. When the tissue that adhered around the spermatic cord was peeled back, a direct hernia was seen protruding from directly above the pubic bone into the scrotum (Figure 1). The sac was partially released and investigated as far as possible into the abdominal cavity, but the operation was completed by radical surgery using a Mesh Plug (DAVOL Inc., Warwick, RI) without it being possible to confirm necrosis of the small bowel.

**Figure 2:**
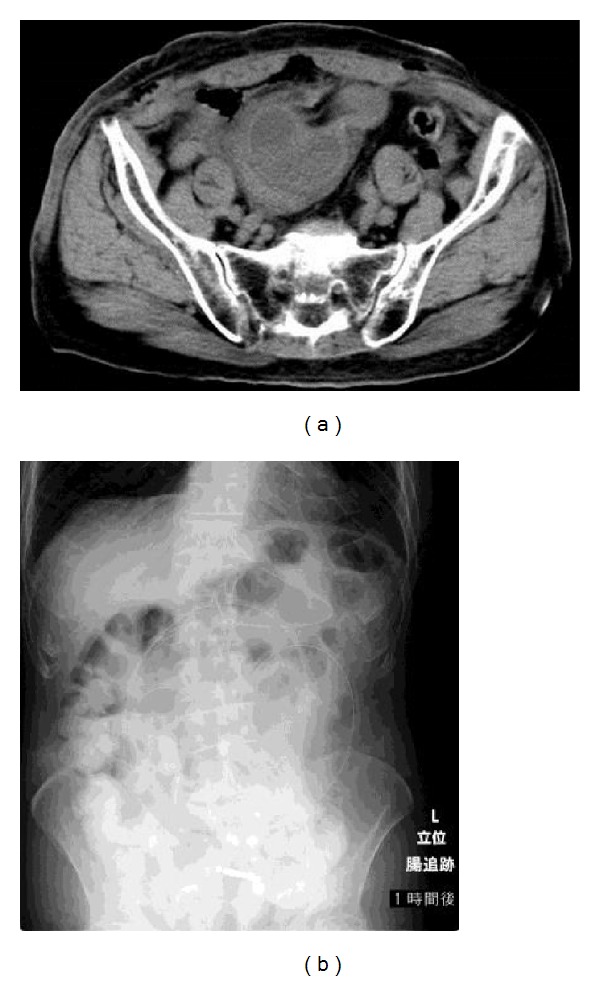
(a) A cyst-like bowel loop is visible in the pelvic cavity. Bowel obstruction is not complete, and localized adhesion of the small bowel is thought to be due to repeated prolapse and incarceration over many years. (b) Abdominal X-ray image 1 hour after injection of contrast agent from the ileus tube. Contrast agent has reached the colon, the small bowel is not dilated, and bowel obstruction has improved.

Although the patient's postoperative clinical course included bowel movements and expulsion of flatus, abdominal CT was done because of fluid drainage from the nasogastric tube and persistence of gas in the small bowel on X-ray. The small bowel was less dilated than at admission, but a portion of it appeared to be tumor-like inside the pelvic cavity (Figure 2(a)). This was thought to be the result of repeated hernial incarcerations over many years that had led the small bowel to adhere globosely. An ileus tube was inserted on the 8th day of admission. Subsequent drainage was <300 mL/day, and there were bowel movements and expulsion of flatus. The ileus tube was removed on the 13th day of admission because ileus tube imaging showed transfer of contrast agent to the colon after 1 hour (Figure 2(b)). From the 14th day of admission, the patient resumed eating and was able to consume an entire meal, and his clinical course was problem-free, but when the diet was increased to 7 : 1 rice gruel on the 20th day of admission, he developed sudden abdominal pain, fever, and symptoms of peritoneal irritation over the whole abdomen. Abdominal CT detected free air, and emergency abdominal surgery was performed (2nd operation) based on a diagnosis of peritonitis due to gastrointestinal perforation.

**Figure 3:**
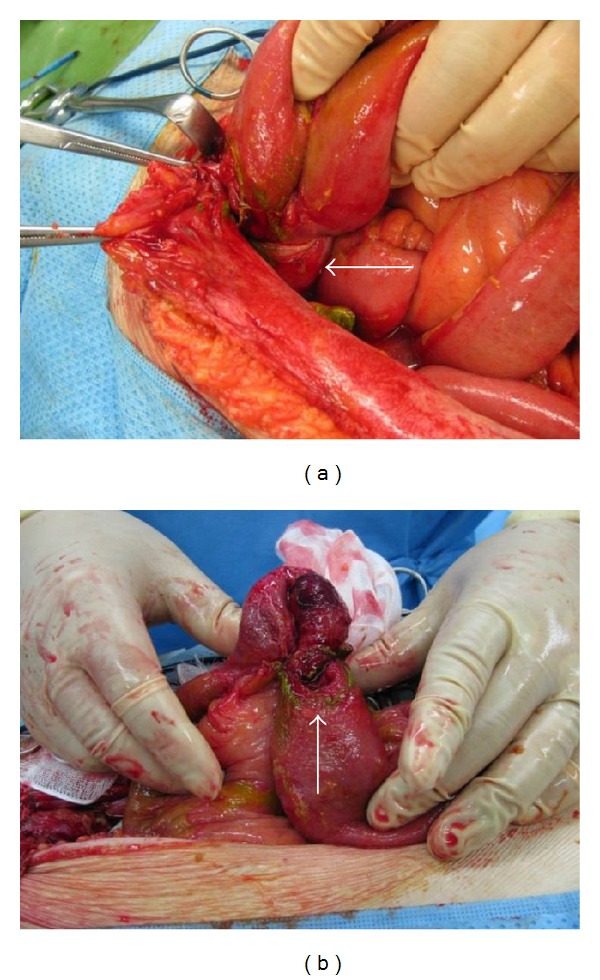
(a) Small bowel in the vicinity of the pubic bone, protruding into the abdominal cavity and surrounded by peritoneum. Each sac containing incarcerated small bowel has undergone reduction into the abdominal cavity. The arrow indicates the hernial sac that has undergone reduction en masse. The small bowel on the oral side has perforated at the point directly before the bowel enters the sac, and a hole that is the size of the head of an index finger has opened up. (b) The arrow shows the perforation in the small bowel after elimination of the reduction en masse.

The lower abdomen was opened by midline incision, revealing the abdominal cavity to be contaminated with intestinal fluids. Tracing the dilated small bowel, thickened peritoneum in the vicinity of the left internal inguinal ring was found to be protruding into the abdominal cavity, and the small bowel was pinched at the same site and perforated on the dilated oral side directly before entering the hernial sac (Figures 3(a) and 3(b)). It was thought that reduction en masse of the incarcerated hernia had taken place during reduction, causing perforation of the small bowel. There was no necrosis of the incarcerated small bowel. The perforated portion of the small bowel was resected, and the internal inguinal ring was closed by suture from the abdominal cavity. There were no notable postoperative complications, and on the 6th day of admission after the 2nd operation, the patient resumed eating and was discharged.

## 3. Discussion

As surgeons, we often encounter incarcerated inguinal hernia as part of our daily practice. Complications of manual reduction include damage to the hernia contents and reduction of necrotic bowel, but hernial reduction en masse is less widely known. It is a rare condition in which the hernial sac is returned to the properitoneal space along with incarcerated bowel. The condition requires surgical reduction to release strangulation. However, reduction en masse can be difficult to diagnose because of generalized and nonspecific symptoms. The first report of reduction en masse was by Luke in 1843 [1]. According to Pearse, reduction en masse occurs in about 1 in 13,000 hernia cases [2]. About 200 cases have been reported worldwide [3], but the first Japanese case report was in 1990, and since then, only 15 cases have been reported. However, rather than this being a particularly rare condition, the true number of cases is probably much higher because the condition has not been well known until around 2008, when reports started increasing. Reduction en masse occurs almost exclusively in the setting of chronicity where the hernia has been repeatedly reduced on several separate occasions [4]. This repeated manipulation may lead to fibrosis of the hernial sac, causing the sac to develop areas of narrowing and to become unyielding, which prevents the bowel loops from freely sliding back into the abdomen [5]. There is usually a history of difficult reductions, the last being more difficult, after which the symptoms of intestinal obstruction fail to subside or subside only temporarily [6, 7]. Computed tomography shows “the properitoneal hernia sac sign,” which is defined as “presence of a hernia sac in the properitoneal space (and not in the inguinal/femoral canal) containing an obstructed/incarcerated bowel loop and causing small bowel obstruction” to identify “reduction en masse of inguinal hernia” [4]. Hernial incarceration requires an accurate preoperative diagnosis, working on the assumption that strangulation is involved.

What is notable about this case is that reduction en masse occurred in a Zollinger classification type VII (combined type) hernia [8] and that reduction en masse was not detected until gastrointestinal perforation 20 days after the first operation, since it was assumed that surgery had cured the hernia, and bowel obstruction due to incarceration was not complete. Reduction en masse occurred during reduction of a recurrent hernia during an outpatient visit, but it was not detected at that time, and because it was a recurrent case involving adhesions, the emergency surgery conducted that day only treated the direct hernia that was confirmed during surgery. In fact, the indirect hernia caused the incarceration, and its reduction led to the reduction en masse. This patient had previously undergone reduction of hernial incarceration as an outpatient, and radical surgery by the anterior approach was chosen because, being directly after reduction, there was no prolapse in the inguinal region. However, because the indirect hernia's sac was not in the inguinal region, this hernia was not noticed at surgery, and treatment during surgery was only given for the direct hernia, which was unrelated to the incarceration in the inguinal region. Furthermore, although there were mild symptoms of bowel obstruction, obstruction was not complete, and therefore passage obstruction was improved by pressure reduction, and the patient began eating with the reduction en masse intact. The small bowel then perforated after resumption of eating.

The presence of reduction en masse might ideally be checked by CT or ultrasound after reduction if available, particularly in cases of incarceration and self-reduction over a long period, because there exist, although it might be a rare case, very mild symptoms of bowel obstruction due to reduction en masse. When reducing an incarcerated hernia, the clinician must be aware of the possibility of reduction en masse, in addition to damage to the hernia contents and necrosis. Especially, when performing an anterior hernia repair, check for a second hernia. When in doubt, and if possible, the surgeon can always open such a large hernia sac as shown here and palpate from inside. Otherwise, severe symptoms of undiagnosed reduction en masse can occur after many weeks.
